# Anti-inflammatory activity of extracts of Bushen-Qiangdu-Zhilv decoction, a Chinese medicinal formula, in M1-polarized RAW264.7

**DOI:** 10.1186/1472-6882-14-268

**Published:** 2014-07-28

**Authors:** Run-Yue Huang, Jie-Hua Lin, Xiao-Hong He, Xiong Li, Chuan-Li Lu, Ying-Yan Zhou, Jun Cai, Yi-Ting He

**Affiliations:** Department of Rheumatology, The Second Affiliated Hospital, Guangzhou University of Chinese Medicine (Guangdong Provincial Hospital of Chinese Medicine), Guangzhou, 510006 China; Central Laboratory, The Second Affiliated Hospital, Guangzhou University of Chinese Medicine (Guangdong Provincial Hospital of Chinese Medicine), Guangzhou, 510006 China; Department of Cerebral Surgery, The Second Affiliated Hospital, Guangzhou University of Chinese Medicine (Guangdong Provincial Hospital of Chinese Medicine), Guangzhou, 510006 China

## Abstract

**Background:**

Bushen-Qiangdu-Zhilv Decoction (BQZ) is one of famous traditional Chinese medical formula for treating ankylosing spondylitis (AS). However, the mechanisms underlying effects of BQZ remains unknown. Pro-inflammatory cytokines, tumor necrosis factor (TNF)-α and interleukin (IL)-1, play an important role in AS. We therefore evaluated if BQZ could affect the expression of these cytokines.

**Methods:**

Crude extracts were prepared and fractioned with petroleum ether (PE), ethyl acetate (EA), n-butanol (BU) and finally water (ACE). The stability of the extracts was confirmed by high-pressure liquid chromatography (HPLC) analysis. M1-polarized RAW264.7 was induced and subsequently treated with BQZ extracts. Quantitative real-time PCR experiments were performed to measure mRNA expression of TNF-α and IL-1.

**Results:**

It was found that TNF-α could be significantly suppressed by ACE extracts, whereas IL-1 was dramatically inhibited by BU extracts, which was further confirmed by dose-dependent experiments. Importantly, MTS assays showed that both ACE and BU extracts had a low cytotoxicity.

**Conclusion:**

Altogether, our study indicates that BQZ decoction exerts anti-AS effects via its anti-inflammatory activity and may have a low side-effect. Further analysis of the extracts of BQZ decoction could lead to a discovery of some novel drugs adding to therapeutic strategy for AS patients.

## Background

Ankylosing spondylitis (AS) is a systemic inflammatory disease characterized by chronic inflammation of the axial skeleton, the peripheral joints, enthuses as well as the attachments of ligaments [1]. The prevalence of AS is 0.20%–0.54% among Han-Chinese population, which is similar to the prevalence in Europe and America [2]. Regarding therapeutic approaches aimed to treat AS, nonsteroidal anti-inflammatory drugs (NSAIDs) have been considered as the cornerstone of treatment for AS, but still, they are not effective in some cases. Disease-modifying antirheumatic drugs (DMARDs), such as sulfasalazine and methotrexate, are only recommended for treating AS with peripheral arthritis or extra-articular features [3, 4]. However, in cases of exclusive spinal involvement that do not respond to NSAIDs, the merely option is to adopt anti-tumor necrosis factor (TNF) agents [4]. Because anti-TNF therapy suppresses the immune system [5], serious infections are the most frequently reported adverse events of interest across indications for the anti-TNF drugs [6]. In addition, given the role of TNF in mediating tumor growth [5], the risk of malignancy with anti-TNF therapy has been a concern [7], and there is substantial evidence that the chronic inflammation inherent in the conditions treated with anti-TNF therapy is itself associated with an increased potential for malignancy [6, 8].

Fortunately, traditional Chinese medicine provides an alternative, or better, choice for AS patients. For example, traditional Chinese medical treatment, while effective in treating rheumatoid arthritis, appears to be less effective than Western medical treatment in controlling symptoms, but traditional Chinese medical treatment is associated with fewer side effects [9]. Bushen-Qiangdu-Zhilv Decoction (BQZ) was established by Prof. Shu-De Jiao who is a well-known traditional Chinese medicine master in Rheumatology. Modified BQZ decoction has been demonstrated to be more effective than sulfasalazine, a typical DMARD for treating AS, in relieving clinical symptoms and signs as well as inflammatory activity indicators of AS patients [10]. However, the mechanisms underlying BQZ decoction effects remains unclear. Herein, in this study, the crude extracts of BQZ decoction was prepared and fractioned, and effects of crude extracts of BQZ decoction on pro-inflammatory cytokines, TNF-α and interleukin (IL)-1, were determined.

## Methods

### Cell lines and cell culture

RAW 264.7 macrophage-like cell line was ordered from American Type Culture Collection (Rockville, MD, USA) and cultured in Dulbecco’s Modified Eagle’s Medium supplemented with 10% fetal bovine serum (FBS). To induce M1-polarized RAW264.7, 100 ng/ml interferon (IFN)-γ was added to cultures for 24 h prior to stimulation with crude extracts of BQZ decoction. Cell lines were propagated at 37°C in an atmosphere of 5% CO_2_.

### Extraction and separation

Bushen-Qiangdu-Zhilv (BQZ) formula is composed of 22 species of herbal plants. All components, purchased from KANGMEI pharmaceutical Co., LTD (Guangzhou, China), were identified by our authors (Prof. Yi-Ting He and Dr. Xiao-Hong He). The formula of BQZ is described in Table 1.

**Table 1:** **The components of BQZ formula**

Components of formula	Quantity
Rhizoma Drynariae	18 g
Fructus psoraleae	12 g
Radix Rehmanniae Praeparata	15 g
Herba epimedii	12 g
Notopterygium root	12 g
Rhizome cibotii	30 g
Radix angelicae pubescentis	10 g
Dipsacus root	18 g
Eucommia ulmoides	20 g
Medicinal cyathula root	12 g
Herba lycopi	15 g
Cassia twig	15 g
Rhizome anemarrhenae	15 g
Radix aconiti carmichaeli (Cooked and sliced)	12 g
honey-fried herba ephedrae	6 g
Rhizome zingiberis	6 g
Rhizoma atractylodis macrocephalae	10 g
Radix clematidis	15 g
Radix Saposhnikoviae	12 g
Raw semen coicis	30 g
Root of common peony	12 g
Radix paeoniae alba	12 g

Total 352 g of BQZ formula was boiled with 1.5 L ultrapure water in a Chinese medicine decocting pot (Guangzhou WEN XIN electronics co., LTD., China) for 2 h, yielding final 400 ml of solution. The obtained solution was filtered and subsequently dried using a centrifugal evaporator (Genevac Ltd., UK) for 48 h, following evaporated in a rotavapor (IKA laboratory, Germany) at 25°C. Finally, 6.4 g crude extract of BQZ decoction was obtained.

6.4 g of the crude extract were suspended in 400 ml ultrapure water and the solution was extracted three times with 500 ml of solvents of different polarity starting with petroleum ether (PE), ethyl acetate (EA), n-butanol (BU) and finally water (ACE). The obtained fractions were evaporated to dryness yielding 0.09 g petroleum ether, 0.72 g ethyl acetate, 0.549 g n-butanol and 4.50 g water fraction. High-pressure liquid chromatography (HPLC) analysis was performed as previously described [11]. To observe which fractioned extracts is responsible for the BQZ effects, 50 μg/ml of these crude extracts were used to stimulate M1-polarized RAW264.7 for 24 h.

### Flow cytometric analysis

The M1 polarized RAW264.7 was washed with PBS, and cells were subsequently stained with FITC-conjugated antibody, directed against cell surface marker CD86 [12], or with corresponding isotype controls for 20 min at 4°C. Cells were analyzed using FACSCalibur (Becton Dickinson, Erembodegem, Belgium). Data were analyzed using fluorescence-activated cell sorting (FACS) analysis and shown as mean fluorescent intensity (MFI).

### Reverse transcriptase and quantitative real-time PCR

Total RNA was extracted using Trizol reagent (Invitrogen, Grand Island, NY), and cDNA was subsequently synthesized from 2 μg of total RNA using a high capacity cDNA reverse transcription kit (Promega, Madison, WI), according to the manufacturer’s instruction. Quantitative real-time PCR was performed using gene-specific primers and SYBR Green qPCR SuperMix (Bio-Rad Laboratories, Inc. Berkeley, CA). The following primer sequences were used: GAPDH, (forward) 5′-GTTTTCAGGGATGAAGCGGC-3′and (reverse) 5′-TTTGTCAAGCTCATTTCCTGGTATG-3′; TNF-ɑ, (forward) 5′-GTGTCCCAACATTCATATTGTCAGT-3′and (reverse) 5′-TGGGAAGAGAAACCAGGGAGA-3′; IL-1, (forward) 5′-TGGGATAGGGCCTCTCTTGC-3′and (reverse) 5′-CCATGGAATCCGTGTCTTCCT-3′; arg1, (forward) 5′-TACAAGACAGGGCTCCTTTCAG-3′and (reverse) 5′-TGAGTTCCGAAGCAAGCCAA-3′; iNOS, (forward) 5′-TGAGTTCCGAAGCAAGCCAA-3′and (reverse) 5′-AGACCTCAACAGAGCCCTCA-3′.

Real-time PCR was performed using the CFX96 Touch Deep Well™ Real-Time PCR Detection System (Bio-Rad) with the following steps: 50°C 2 min, 95°C 10 min, 40 cycles at 95°C 15 s and 60°C 60 s. The expression of target genes in the treatment and control groups was normalized using the house-keeping gene GAPDH and the fold change in the expression of each target gene was calculated by the 2-ΔΔCT method.

### Cytotoxicity assay

Cytotoxicity of ACE and BU extracts was detected by MTS, i.e., CellTiter 96® AQueous One Solution Cell Proliferation Assay, according to the manufacturer’s instruction (Promega). Briefly, cells were seeded at the same density into 96-well plates and incubated overnight for attaching. After proper treatment, the control and treated cells were incubated for the indicated times. Following adding 20 μl of MTS [3-(4,5-dimethylthiazol-2-yl)-5-(3-carboxymethoxyphenyl)-2-(4-sulfophenyl)-2H-tetrazolium, inner salt] in each micro well, and plate was read using a microplate reader at wavelength of 492 nm (Bio-Rad, Philadelphia, PA, USA).

### Statistical analyses

Data are shown as means ± SD from experiments repeated at least twice. For the comparisons between two groups, Student's t test was utilized. One-way ANOVA followed by Dunnett’s test was employed for comparisons among more than two groups. Statistical analyses were conducted by SPSS 11.6 statistical software (SPSS, Chicago, IL). A two-tailed P value of < 0.05 was considered to indicate statistical significance.

## Results

### In vitro M1 macrophage polarization

**Figure 1:**
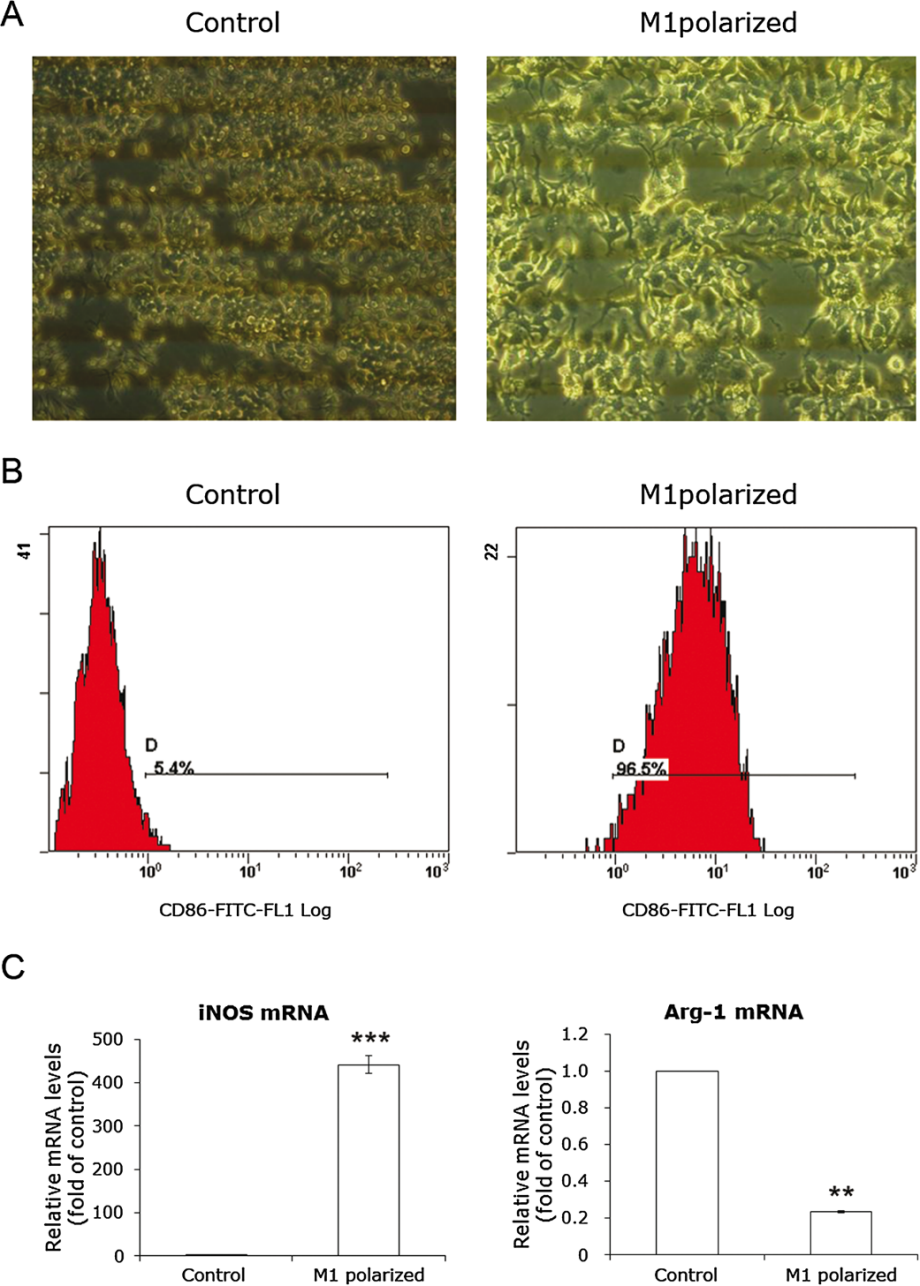
**Induction of M1-polarized RAW264.7.** RAW264.7 was polarized toward the M1 phenotype by stimulating with 100 ng/ml IFN-γ for 24 h. **A**, microscope shots pictures of RAW264.7. The right panel is the M1 phenotype. **B**, flow cytometry detection of CD86, a signature marker of M1 cells. **C**, the relative mRNA levels of iNOS and Arg-1 were determined by real-time PCR. The mRNA levels of these two molecules in RAW264.7 without treatment were used as the controls. Data are presented as mean ± SD of three independent experiments done in triplicate. **p < 0.05 and ***P < 0.001 versus control.

M1 macrophage phenotype was induced on RAW264.7 by stimulating with 100 ng/ml IFN-γ for 24 h (Figure 1A). Flow cytometric analysis were conducted to examine CD86 expression in cell surface, since CD86 is well-known to be a signature marker of M1 macrophage. As shown in Figure 1B, CD86 was expressed in 96.5% RAW264.7 exposed to IFN-γ, confirming M1 polarization of RAW264.7. Real-time PCR further confirmed the data obtained by flow cytometric analysis. The overall polarization of RAW264.7 shifted to M1, as inducible nitric oxide synthase (iNOS) expression increased and arginase (Arg)-1 expression decreased (Figure 1C) [12].

### Effects of crude extracts on TNF-α and IL-1 mRNA expression

**Figure 2:**
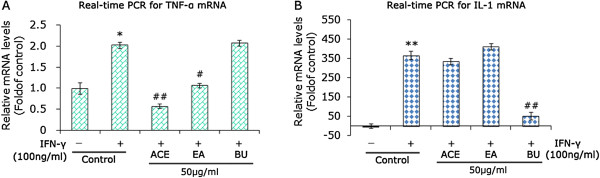
**Effects of fractioned extracts on TNF-α and IL-1 mRNA expression.** Quantitative real-time PCR evaluation for the effects of crude extracts of BQZ decoction on mRNA expression of TNF-α **(A)** and IL-1 **(B)**. M1-polarized RAW264.7 was stimulated with the different crude extracts (50 μg/ml respectively) for 24 h. The M1-polarized RAW2647 without extracts stimulation were used as control and RAW264.7 without treatment were employed as the additional controls. Total RNA was extracted and subjected to real-time PCR experiments. Data are expressed as mean ± SD of three independent experiments done in triplicate. *p < 0.01 and **P < 0.01 versus the additional controls, while #p < 0.05 and ##p < 0.01 versus control.

The crude extracts, including extracts fractioned by PE, EA, BU and finally ACE, were used to treat the M1-polarized macrophages. PE extracts was not used, since cells were totally killed when treating with PE extracts. M1-polarized RAW264.7 was stimulated with 50 μg/ml of these crude extracts for 24 h respectively. M1-polarized RAW264.7 without treatment was used as the controls. Total RNA was extracted from cell lysates and subjected to quantitative real-time PCR experiments. As shown in Figure 2A, ACE extracts significantly suppressed mRNA expression of TNF-α by about 4.0-fold of control with p < 0.01. In addition, EA extracts reduced TNF-α mRNA expression by about 2.0-fold of control (p < 0.05). However, both ACE and EA extracts did not have significant effects on IL-1 mRNA expression (Figure 2B). Interestingly, BU extracts had an opposite effects. The level of IL-1 mRNA, but not TNF-α mRNA was dramatically decreased by BU extracts by more than 7.0-fold of control (Figure 2B, p < 0.01). Herein, it appears that EA extracts are responsible for targeting TNF-α, while BU extracts are accountable for suppressing IL-1 in BQZ decoction. Further experiments were conducted to verify the effects of EA and BU extracts on pro-inflammatory cytokines.

**Figure 3:**
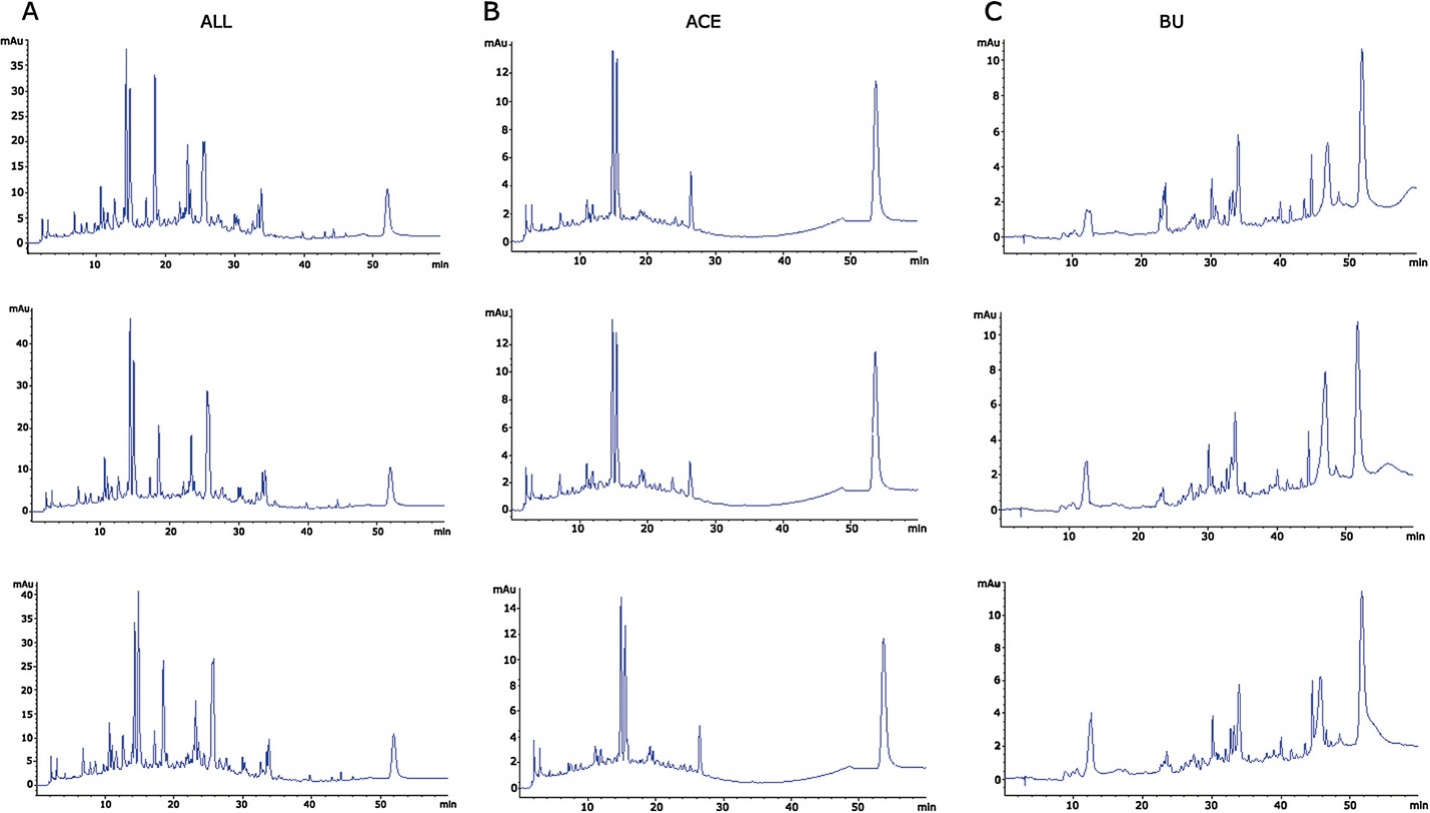
**The stability of the extracts and the preparation technology.** Total crude extracts **(A)**, ACE extracts **(B)** and BU extracts **(C)** was analyzed using a liquid chromatograph. Figures shown were selected from the experiments repeated for four times.

The preparation of crude extracts and the subsequently HPLC experiments were performed more than three times. As illustrated in Figure 3, HPLC chromatograms of total, ACE and BU extracts of BQZ decoction prepared from three independent experiments were quite similar, suggesting the stability of the extracts and the preparation technology.

### ACE extracts suppressed TNF-α expression with a low cytotoxicity

**Figure 4:**
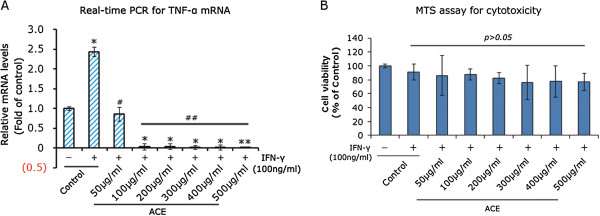
**ACE extracts suppressed TNF-α expression with a low cytotoxicity.** M1-polarized RAW264.7 was treated graded levels of ACE extracts for 24 h. **A**. Real-time PCR evaluation of TNF-α mRNA expression in M1-polarized RAW264.7. RAW264.7 without treatment was used as the additional controls. *p < 0.01 and **P < 0.01 versus RAW264.7 cells without treatment, while #p < 0.05 and ##p < 0.01 versus M1-polarized macrophages without treatment. **B**. The cytotoxicity of ACE extracts was determined by MTS assay, and the results were presented as% of controls. Data are expressed as mean ± SD of three independent experiments done in triplicate.

To confirm the suppressing effects of ACE extracts on mRNA expression of TNF-α in M1-polarized phenotype, RAW264.7 was treated with graded levels of ACE extracts following 24 h stimulation with 100 ng/ml IFN-γ. As shown in Figure 4A, mRNA level of TNF-α was suppressed by ACE extracts in a dose-dependent manner. The expression of TNF-α mRNA was almost completely inhibited when cells exposed to ACE extracts at the concentrations from 100 μg/ml to 500 μg/ml (p < 0.001 respectively).

To determine if the decreased levels of TNF-α mRNA were due to cell death, cells survival was examined by MTS assays. MTS assay is an update method from MTT assay, which is a typical and widely used tool for measurement of cell survival. After stimulation with 100 ng/ml IFN-γ, RAW264.7 was treated graded levels of ACE extracts for 24 h and MTS experiments were subsequently conducted. Surprisingly, ACE extracts did not affect cell survival significantly, even at the concentration of 500 μg/ml (Figure 4B). Our findings suggest that ACE extracts can suppress expression of TNF-α mRNA in M1-polarized RAW264.7 with a low cytotoxicity.

### BU extracts suppressed IL-1 expression with a low cytotoxicity

**Figure 5:**
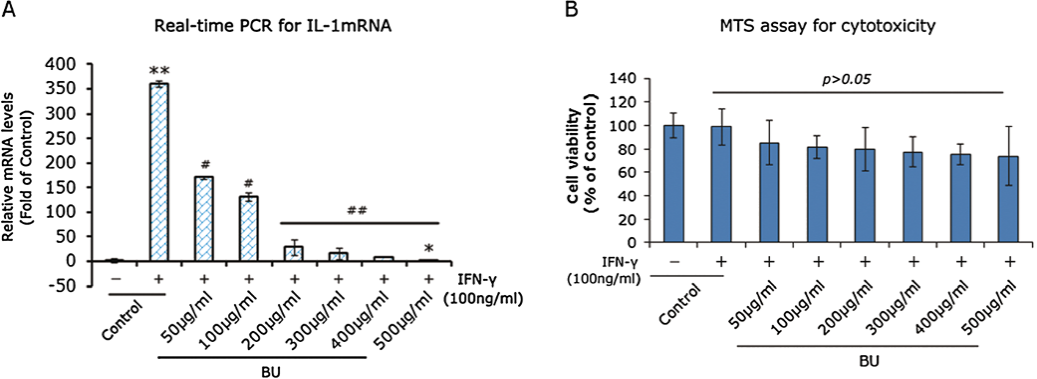
**BU extracts suppressed IL-1 expression with a low cytotoxicity.** M1-polarized RAW264.7 was treated graded levels of BU extracts for 24 h. **A**. Real-time PCR evaluation of IL-1 mRNA expression in M1-polarized RAW264.7. RAW264.7 without treatment was used as the additional controls. *p < 0.01 and **P < 0.01 versus RAW264.7 without treatment, while #p < 0.05 and ##p < 0.01 versus M1-polarized RAW264.7 without treatment. **B**. The cytotoxicity of BU extracts was determined by MTS assay, and the results were presented as% of controls. Data are expressed as mean ± SD of three independent experiments done in triplicate.

RAW264.7 was treated with increasing concentrations of BU extracts following 24 h stimulation of 100 ng/ml IFN-γ. Figure 5A showed that BU extracts could dose-dependently inhibit IL-1 mRNA expression. 50 μg/ml BU extracts significantly suppressed IL-1 mRNA expression (p < 0.05), which is in line with the data shown in Figure 2B. In addition, BU extracts totally abrogated the increased expression of IL-1 mRNA in M1-polarized RAW64.7 to the level under control (p < 0.001). MTS assays demonstrated that increasing concentrations of BU extracts could not kill M1-polarized RAW2647, albeit there was an inhibitory trend (Figure 5B). Together, these data strongly suggest that BU extracts is able to suppress IL-1 expression with a low cytotoxicity.

## Discussion

Macrophage activation plays an important role in the pathology of AS [7, 10, 13]. Macrophages are a population of cells derived from CD34 positive bone marrow progenitors, from which blood pro-monocytes are differentiated and developed into monocytes which extravasate into tissues where they become “resident” tissue macrophages [14]. Macrophages may be recruited and subsequently activated as consequence of any local disturbance of tissue homeostasis, such as infections, immune response and malignancy [7, 15]. Activated macrophages, also known as polarized macrophages, present different phenotypes, M1 (classical activation) and M2 (alternative activation) in general, in relation to the nature of the recruiting stimulus and the location [7, 16]. These two polarized phenotypes are considered to exhibit opposing activities, being either polarized towards pro-inflammatory or anti-inflammatory activity. Many pro-inflammatory cytokines, including TNF-α and IL-1, are derived from M1-polarized macrophages, and importantly these cytokines are associated with AS [17, 18]. Moreover, M1-polarized macrophages have been demonstrated to be expanded in AS patients [5]. Mouse macrophage-like cell line RAW264.7 is, to some extent, an ideal macrophage model for in-vitro studies [19]. Therefore, in this study, RAW264.7 was used as an in-vitro model, and M1 phenotype was polarized by exposure to IFN-γ [19].

Biological agents targeting inflammatory cytokines such as TNF-α have widely used in recent years as effective medications for treating AS, while numerous cases of the appearance of malignant tumors in patients receiving these drugs have been reported [20]. In addition, not all AS patients achieve remission or a major clinical response to NSAIDs and TNF-α blockers [21]. Though biological agents targeting IL-1, anakinra for example, has proven to be well tolerated and indicated in the treatment of rheumatoid arthritis, the data in AS are still lacking. Therefore, are there any alternatives?

BQZ decoction is a famous Chinese medicine formula with a long history for application in the treatment of AS. Crude extracts was prepared from BQZ decoction and subsequently fractioned. The effects of different fractioned extracts on the expression of pro-inflammatory cytokines were screened. Strikingly, in M1-polarized RAW264.7, ACE extracts could significantly suppress mRNA level of TNF-α, whereas BU extracts dramatically inhibited IL-1 mRNA expression. These findings suggest that BQZ decoction could be a natural antagonist to pro-inflammatory cytokines. To confirm this data, concentration-dependent experiments were conducted subsequently. The discovery of that TNF-α mRNA expression could be totally inhibited by ACE extracts suggests that BQZ decoction can be an alternative medication in AS patients intolerance to TNF-α blockers [21].

It is well-known that chronic inflammation in AS can lead to extensive new bone formation throughout the spine [22], and importantly, IL-1 may result in stimulation of bone formation [23, 24]. The data that expression of IL-1 mRNA was completely blocked by BU extracts implies that BQZ decoction is capable of relieving new bone formation. Therefore, BQZ decoction might be a better medication than many other biological agents targeting TNF-α, such as infliximab, etanercept, adalimumab, as the treatment of these agents does not halt new bone formation [25].

In addition, we tested if the decrease of TNF-α and IL-1 was due to the death of cells. Herein, MTS assays were conducted and the results turned out to be negative. Both ACE and BU extracts could not induce cell death in M1-polarized RAW264.7, suggesting the low cytotoxicity of the extracts of BQZ decoction. Considering modified BQZ decoction was more efficacious than sulfasalazine [10], the lower cytotoxicity of the extracts of BQZ decoction suggests that, compared with those Western medications, traditional Chinese medications could be a safer and better choice for treating AS.

## Conclusion

In summary, with the low cytotoxicity, crude extracts of BQZ decoction fractioned with ACE and BU could block TNF-α and IL-1 mRNA expression in M1-polarized RAW264.7 respectively, suggesting that BQZ decoction could be an better and alternative medication for treating AS patients. Further analysis of fractioned extracts of BQZ decoction may lead to some novel drugs for treating AS with more efficacious and low toxicity.
